# Maternal Corporeal Support in Terminal Stage Brain Astrocytoma: A Case Report and Literature Review

**DOI:** 10.3390/healthcare14081055

**Published:** 2026-04-15

**Authors:** Eleni N. Sertaridou, Emmanouela Tsouvala, Vasilios E. Papaioannou, Christina Alexopoulou

**Affiliations:** 1 Medical School, Democritus University of Thrace, 68100 Alexandroupolis, Greece; esertari@med.duth.gr (E.N.S.); vapapa@med.duth.gr (V.E.P.); 2 ICU Department, University Hospital of Alexandroupolis, 68100 Alexandroupolis, Greece; emtsouvala@gmail.com

**Keywords:** brain death, pregnancy, maternal, astrocytoma, gliomas, intensive care unit, brain cancer

## Abstract

**Background**: The care and management of a pregnant woman suffering from end-stage brain cancer is surrounded by medical, legal, and ethical controversies. When this brain pathology leads to brain death (BD), continuation of life-sustaining treatments has been considered futile and unethical. An exception could be the case of pregnancy, in order to deliver a healthy neonate. **Aim**: The presentation of a pregnant woman with a terminal stage brain astrocytoma, admitted in the intensive care unit (ICU) to support the pregnancy, until optimal fetal viability, after she had neurological deterioration and confirmed BD, and a brief literature review of previously relevant published cases. **Case Presentation**: A 36-year-old woman with a medical history of brain astrocytoma in the last 2 years was admitted in ICU for the first time due to status epilepticus, six months after she stopped anticonvulsant therapy. Her epilepsy was controlled, and a pregnancy of 14 weeks was confirmed. Two weeks later, she deteriorated. After a multidisciplinary approach, it was decided to mechanically ventilate the patient and support the pregnancy. Brain death was determined after a couple of days. **Results**: A cesarean section was performed 11 weeks after BD diagnosis (at 27 weeks of gestational age) resulting in the delivery of a live, premature infant, weighing 549 gr. **Conclusions**: Maternal corporeal support can maximize the chances for survival in the neonate by prolonging the pregnancy of a brain-dead woman.

## 1. Introduction

Gliomas of astrocytic origin are the most common Central Nervous System tumors in persons over 40 years old, accounting for 22.9% of all tumors [1], more frequently seen in males than females, with a sex ratio of 1.4:1 [2,3]. However, gliomas are the most prevalent histological type of primary malignant brain tumor in women, with an annual occurrence of 2.6 in 100,000 women in the United States [4]. These brain tumors are very aggressive, with a very poor prognosis [2]. The median progression-free and overall survival have been reported at only 7.4 months and 15 months, respectively [5,6], while the latest findings suggest that astrocytomas seem to be hormone-sensitive tumors [2,7].

Cancer during pregnancy is uncommon, occurring approximately once per 1000 pregnancies annually, corresponding to 0.07% to 0.1% of all malignant tumors [8]. The most common malignancies associated with pregnancy are, in order of decreasing frequency, melanoma and breast cancer, cervical cancer, lymphomas and leukemias [8]. The reported incidence of primary brain tumors in pregnant women is slightly lower, but the relative frequencies of each brain tumor type appear to be similar for pregnant and nonpregnant women [9]. The occurrence of astrocytomas in pregnancy is considered rare, and the exact incidence is difficult to determine, since there is a small number of literature reports, including only small case series [9,10] and a few isolated case reports [4,11,12,13,14,15] (Table 1). Although there is limited data specifically focusing on astrocytomas during pregnancy, retrospective studies in glioma behavior suggest that both pre-existing tumor volume and growth increased during pregnancy [16], changes that were correlated with clinical deterioration and seizure emergence, due to hormonal fluctuations and increased intracranial pressure [17,18,19].

Pregnancy in a woman with end-stage brain cancer is an occasional condition. When the neurological status of the patient is further deteriorated and the patient is declared brain dead (BD), many ethical, legal, and clinical questions emerge [24,25]. The complexity of BD pathophysiology in pregnancy requires a multidisciplinary approach, and the corporeal support of the BD mother can lead to significant complications that eventually affect embryonic growth [26,27]. Brain-dead patients experience a multitude of physiological insults resulting in a loss of essential homeostatic brain functions, which could manifest with diabetes insipidus, panhypopituitarism, hemodynamic instability, systolic myocardial dysfunction, cardiac arrhythmias, infections, disseminated intravascular coagulation, and hypoxemia [23]. Successful prolongation of pregnancy in such patients, to increase the chance for fetal survival, could represent a significant challenge in the area of intensive care medicine, obstetrics, and medical ethics.

Herein, we describe the case of a 36-year-old pregnant woman suffering from astrocytoma, who was corporeally supported in the ICU, after she had neurologically deteriorated, to maintain the pregnancy. The singularity and uniqueness of this case is the combination “pregnancy”—“astrocytoma”—“brain death”—“maternal corporeal support”, since according to the results of this literature review, the majority of cases of BD during pregnancy, that have been reported, referred to benign acute brain catastrophic lesions (Table 2), such as brain ischemia [28], postanoxic encephalopathy [25,27,29,30], subarachnoid hemorrhage due to aneurysm [24,31,32] or arteriovenous malformation rapture [33] and intracerebral hemorrhage [34]. In addition, the majority of brain tumor cases that existed in the literature (Table 1) referred to patients who were initially diagnosed with a brain tumor during pregnancy and were treated without requiring prolonged intensive multiorgan support [10,13,15].

The purpose of this report is primarily to present the unusual case of a pregnant woman with a known end-stage astrocytoma who was decided to be corporeally supported after she had neurologically deteriorated to maintain the pregnancy. Secondly, we aim to emphasize all ethical, legal, and clinical issues that emerged through a brief literature review.

## 2. Methods

**Figure 1:**
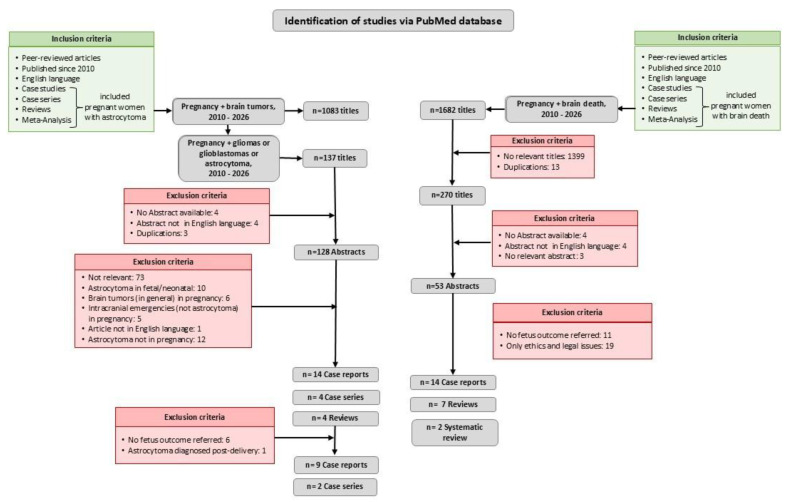
Flow diagram. This review included peer-reviewed articles in English, published since 2010, in PubMed. It contained all existing kinds of original clinical research (case reports and case series) and a few relevant reviews, principally referring to “astrocytoma in pregnancy” and to “brain death in pregnancy”. Through this review, no title referring to “brain death due to astrocytoma in pregnancy” was found.

This is a case report presentation and a brief review that critically examines the recent literature indexed on Pub-Med since 2010 using the following key terms: “Astrocytoma”, “Gliomas”, “Pregnancy”, “Maternal”, “Intensive Care Unit”, “Brain tumors” and “Brain death”. The search was limited to peer-reviewed articles published in English, involving clinical cases or case series, and a few reviews that contained a notable number of relevant cases. After screening the titles for possible relevance, and excluding duplicates, 128 abstracts were reviewed; 4 abstracts were not available, 4 were not in the English language and 3 were duplicated. Finally, 14 case reports, 3 case series and 4 reviews that referred to brain astrocytoma in pregnancy were included in the study, since from the 128 abstracts 104 were not relevant and 1 was not in English language. Moreover, from the 53 abstracts that were reviewed, finally 14 case reports, 7 reviews and 2 systematic reviews that referred to brain death in pregnancy were included in the study. The references from the included articles were also reviewed for additional potentially relevant papers (Figure 1). The literature review focused on ethical, legal, and clinical issues that emerged during the patient treatment.

## 3. Case Presentation

**Figure 2:**
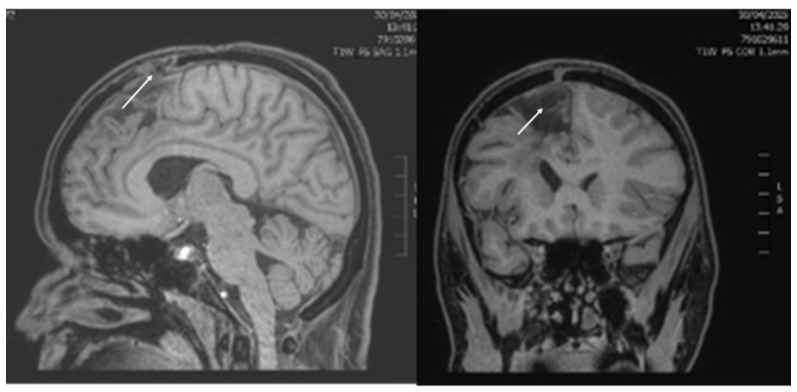
Brain MRI (T1-weighted images) upon the initial admission to the ICU due to status epilepticus. Right parietal lobe and corpus callosum postsurgical porencephaly with peripheral gliosis, and a small heterogenous high signal mass, indicating possible tumor recurrence (marked with white arrow).

A 36-year-old with a past medical history of astrocytoma in the last 2 years, who was 14 weeks pregnant, presented to the emergency department (ED) of the University Hospital of Alexandroupolis suffering from seizures. She was treated with levetiracetam, lacosamide, and benzodiazepines, without managing to control seizures. Twenty minutes later, she was sedated, endotracheally intubated, and transferred to the Intensive Care Unit (ICU) due to status epilepticus. Meanwhile, the brain Magnetic Resonance Imaging (MRI) revealed recurrence of the pre-existing tumor (Figure 2).

**Figure 3:**
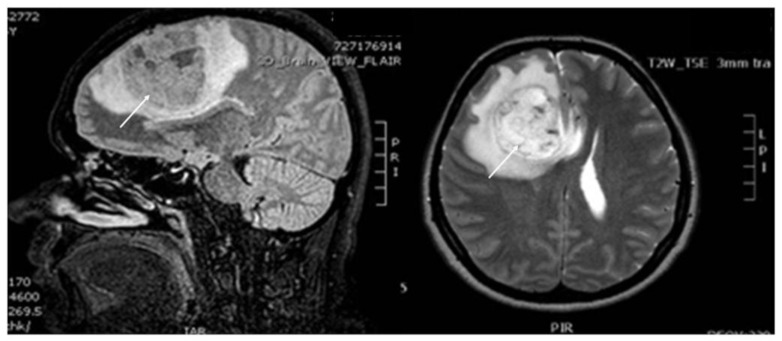
Preoperative brain MRI of the brain at the time of the first diagnosis, 24 months earlier. A heterogenous high signal mass in the right parietal lobe (marked with white arrow), with perifocal edema effacing adjacent sulci, pressure on the right lateral ventricle and shift of the midline to the left including the genu of corpus collusion (Left: FLAIR image, right: T2-weighted image).

According to the patient’s medical history, she was first diagnosed with an astrocytoma stage IV 24 months ago (Figure 3). She was then operated on and treated with postsurgical adjuvant radio- and chemotherapy. Eighteen months later, she was free of symptoms, and the brain Computed Tomography (CT) scan revealed adequate control of the disease. Therefore, she stopped the anticonvulsant therapy and became pregnant, although she and her family were informed of the poor prognosis of her cancer and the possible rapid deterioration in case of pregnancy.

Six months after antiepileptic drug interruption, she was transferred to the ED for the first time and was intubated due to status epilepticus and admitted in the ICU. Anticonvulsant therapy was titrated, and she was extubated after 3 days with a normal Glasgow Coma Scale (GCS) score. Her pregnancy appeared to be without any complications, since the gynecological examination and pregnancy ultrasound showed a normal fetal heart rate, size, and weight at 14 weeks of gestational age. After she was discharged from the ICU, she and her family expressed the wish to maintain the pregnancy and somatically support the patient in case of a future neurological deterioration. A multidisciplinary meeting with intensivists, neurosurgeons, neurologists, neonatal ICU specialists, obstetricians, and legal and bioethics workers was organized. The neurosurgeons and neurologists agreed about the lethal prognosis and the absence of therapeutic options. The obstetricians and neonatal ICU specialists unanimously declared that this was a high-risk pregnancy with minimal possibilities of the birth of a vital neonate. However, a clear legal framework in Greece does not exist. Hence, it was decided to support the pregnancy in case of future deterioration of the mother.

**Figure 4:**
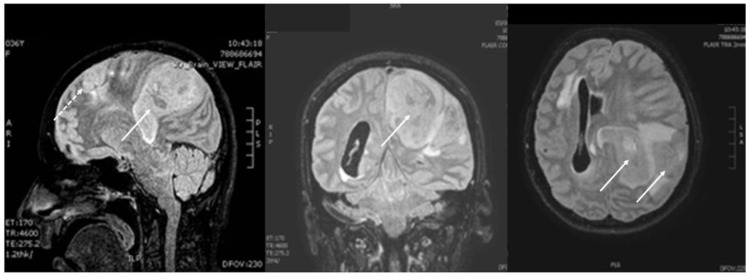
Brain MRI (FLAIR images) upon the second admission to ICU due to neurological deterioration: recurrence of the primary tumor in the right parietal lobe (marked with dotted white arrow) and a large new mass in the same lobe (marked with solid white arrow), deforming the right lateral ventricle down to the brain stem.

Fifteen days later, the patient was transferred to the ED for the second time because of neurological deterioration and respiratory failure. Upon ED admission, she was unresponsive, with a GCS score of 3 (E 1, V 1, M 1), dilated, non-reactive pupils (5 mm), hypoxemic with an oxygen saturation below 92% on air, and bradypnea with a respiratory rate of 6 breaths per minute. She was hypertensive with a blood pressure of 178/95 mm Hg and bradycardic with a heart rate of 43 beats/min, while her core body temperature was normal (36.7 °C). The patient’s family persisted in supporting the patient in order to maintain the pregnancy and bring the fetus to viability. Hence, she was intubated, a brain MRI was performed (Figure 4) and she was transferred to the ICU.

On the day of ICU admission, a central venous catheter, a radial arterial line and a nasogastric feeding tube were placed. Her oxygen saturation, pH and pCO_2_ were rapidly restored on a mechanical ventilator, with a low fraction of inspired oxygen (FiO_2_: 30%), a positive end expiratory pressure (PEEP) of 5 cm H_2_O, a respiratory rate of 10 breaths/min), and a tidal volume (VT) of 350 mL/breath. Her hypertension and bradycardia were restored with a low dose of propofol (100 mg per hour) and remifentanil (2–3 mg per hour) continuous infusion. Her lungs and cardiac examination, clinically and ultrasonographically, were unremarkable, though cefuroxime was administered as a prophylactic antibiotic due to a high probability of aspiration. She rapidly developed diabetes insipidus, and she was treated with vasopressin and crystalloids to maintain a mean blood pressure of 65–70 mm Hg, a mean diuresis rate of 100 mL per hour, and normal serum sodium levels (135–145 mmol/L).

Her pregnancy appeared uncomplicated on a transabdominal ultrasound, since it revealed a fully formed fetus, with a heart rate of 200 beats per minute, normal fetal movements and average size (crown–rump length—CRL of 11 cm, biparietal diameter-BPD of 32 mm, head circumference—HF of 110 mm, abdominal circumference—AC of 102 mm and femur length—FL of 21 mm) and weight (75 gr). According to these parameters, the fetus had a normal growth (10th percentile of intrauterine growth curves) at 16 weeks of gestational age, while the amniotic fluid volume was approximately 120 mL, with an amniotic fluid index (AFI) of 7 cm (normal values 8–18 cm).

Twenty-four hours later, sedation and analgesics were interrupted. She was unresponsive, the pupils were dilated (6–7 mm) and non-reactive, while the brainstem reflexes were abolished. The apnea test was not performed in order to avoid possible negative effects of maternal hypercapnia on the developing fetus. A brain and neck CT angiography or a nuclear medicine scan was also avoided to protect the fetus from radiation. However, the transcranial Doppler showed cerebral circulatory arrest with characteristic oscillatory flow and systolic spikes in major arteries, indicating BD.

With the unconfirmed diagnosis of BD, endocrinology was consulted for the management of adrenal insufficiency and central hypothyroidism. Hydrocortisone 50 mg IV every six hours and levothyroxine 62.5 mcg daily were prescribed and gradually increased over a period of 10 days to 125 mcg, according to the free T4 serum levels. Of note, at the 26th week of gestation, 2 doses of 12 mg dexamethasone, within 24 h, was administered additionally for fetus lung maturation because of the increased risk of emergency premature labor. Her hyperglycemia was treated with subcutaneous insulin, to maintain a blood sugar level of 120–150 mg/dL. Arterial blood carbon dioxide was maintained in the normal pregnancy range of 32–35 mm Hg. Her nutritional requirements were calculated based on the basal energy expenditure in pregnancy, i.e., around 2200–2400 kcal per day, met through the enteral route. Continued multivitamin, folic acid, and trace element support were given daily. Additionally, 4 gr MgSO_4_ was infused when the emergency labor was decided for fetal neuroprotection. Low molecular weight heparin was administered subcutaneously for deep vein thrombosis prophylaxis and was titrated according to daily Anti-Xa levels, which were recorded within normal ranges for thromboprophylaxis (0.2–0.4 IU/mL). Corp’s temperature was continually monitored and regulated at normal ranges through an extracorporeal, non-invasive temperature management system.

The viability of the fetus was monitored with daily fetal heart tone assessment, while weekly ultrasonographic examinations were performed, with biometry of the fetus, to evaluate its growth and rule out any organ development malformation.

Upon the tenth day of ICU admission, the patient had increased secretions, fever (38 °C), moderate hypoxemia (PaO_2_/FiO_2_ reduced from 400 at admission day to 320), and a mild hypotension (increased vasopressin demands to maintain the desired mean blood pressure). Chest X-ray scan was avoided; however, a lung ultrasound revealed a lung consolidation (liver-like hypoechoic area) with air bronchogram (bright lines of air in the fluid) and irregular shaggy sign in the right lower lobe. In bronchial secretions and blood cultures, *Accinetobacter baumannii* was isolated, and ampicillin-sulbactam in combination with Fosfomycin was initiated, according to AST, for the treatment of ventilator-associated pneumonia. During the 11 weeks of maternal corporeal support, our patient also developed *Clostridium difficile* colitis that was treated with vancomycin and metronidazole, candiduria and vaginitis due to *Candida albicans* that were treated with amphotericin B, and septic shock due to *Pseudomonas aeruginosa* that was treated with ceftolozane sulfate/tazobactam sodium.

Although our patient had several predisposed factors for vein thrombosis (such as cancer, pregnancy, coma, central vein catheters in femoral vein, etc.) she did not appear to have vein thrombosis. Possible coagulation disorders were monitored daily by standard laboratory tests, such as prothrombin time (PT), normal international normalized ratio (INR), active partial thromboplastin time (aPTT), fibrinogen (Fib) and platelets (PLT) while enoxaparin dose was titrated according to daily anti-Xa evaluation. D-Dimers levels were recorded only a couple of times during the first week of her ICU admission and were within the normal range for the gestational age. Frequently, a thromboelastographic evaluation through ClotPro^®^ was performed. Upon admission, this test confirmed the physiological mild shift toward hypercoagulability due to pregnancy (characterized by normal clot formation times and increased maximum amplitude and a-angle), while in septic shock it shifted to hypocoagulability due to sepsis-induced mild coagulopathy disorders and amphotericin b-induced thrombopenia (increased clot formation time, reduced maximum amplitude and a-angle).

Several multidisciplinary meetings were held with the patient’s family, obstetrics, and neonatologists throughout hospitalization, while somatic support was continued to maintain the viability of the pregnancy. The fetal monitoring was based on daily ultrasound biometry and Doppler.

After 11 weeks since ICU admission (27th week of gestation), the fetal heart rate had significant decelerations. For this reason, an emergent low transverse cesarean section was performed, and a premature, female neonate was delivered, weighing 549 gr (small for gestational age), with an APGAR score of 3 at 5 min. The labor was uncomplicated and a minimal, unworthy of mention, blood loss was recorded, while the patient remained hemodynamically stable during the operation. The infant was transferred immediately to the neonatal ICU, where she was intubated and mechanically ventilated. A couple of hours after the delivery, the neonatal presented with gradually deteriorated hypoxemia due to respiratory distress syndrome, despite the corticosteroid treatment for fetal lung maturation, and after 48 h developed a pneumothorax, which was drained. Moreover, she was febrile and hemodynamically instable. Two weeks later, she experienced neurological deterioration and intraventricular hemorrhage was diagnosed through transcranial ultrasound. It was drained, but 24 h later it was complicated with substantial intracerebral hemorrhage. The infant died after two weeks due to multiple organ dysfunction, despite the advanced support.

**Figure 5:**
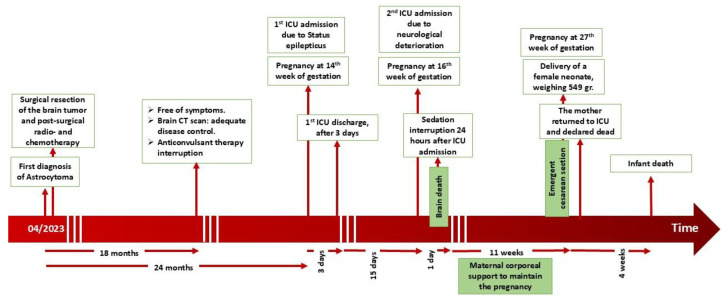
The timeline of the evolution of events.

The mother was transferred back to the ICU. According to brain death/death by neurological criteria (BD/DNC) [31,32], clinical evaluation and apnea testing met the criteria for “brain death”. The patient was declared dead at the time of the completed second apnea test and disconnected from the ventilator (Figure 5).

## 4. Discussion

Astrocytomas are gliomas of astrocytic origin, which are among the most frequent and aggressive primary tumors of the Central Nervous System [2]. According to the 2016 updated WHO Classification of Tumors of the Central Nervous System, based on morphological and immunohistochemical characteristics and molecular signatures, isocitrate dehydrogenase (IDH) mutation and 1p/19q codeletion, astrocytomas are classified into grades, with grade I being the least aggressive pilocytic astrocytoma and grade IV being the most aggressive glioblastoma [39]. The recommended treatment includes a combination of surgery, chemotherapy, and radiotherapy which results in patient outcomes ranging from 18 months of median overall survival in glioblastoma to more than 15 years in 1p19 codeleted oligodendroglioma [2]. Longer overall survival has been reported among women than men, regardless of the molecular status of glioblastoma [40]. This difference in survival was related to chemotherapy efficacy, which was attributed to sex-specific cell cycle and gene-expressed integrin signaling pathway signatures [40]. Our patient was diagnosed with stage IV glioma and underwent surgical resection and postsurgical adjuvant radiotherapy. Eighteen months later, she was free of symptoms, and the brain MRI scan confirmed that the primary tumor remained under control.

### 4.1. Pregnancy and Astrocytoma

Progress in brain tumor treatments and their survival prolongation have led to an increasing number of young women with brain tumors who are deciding on pregnancy [9,10,41]. However, the latest findings converge those gliomas, and more specifically astrocytomas, are hormone-sensitive tumors, since steroids, such as estrogens, progestogens and androgens seem to promote gliomagenesis [2], while pregnancy seems to provoke clinical deterioration, tumor growth, and recurrence [4,19,42].

In the case we present, the patient was diagnosed with an astrocytoma two years ago. The rapid neurological deterioration during the first trimester of pregnancy, 18 months after initial surgical and peri-operative adjuvant treatment, possibly reflects a hormone-sensitive tumor alteration and progression. Yust-Katz et al. [9] retrospectively studied a database of patients who suffered from glial tumors and were pregnant, either at the time of tumor diagnosis or became pregnant during their illness. Of the 23 patients who were included in the study and became pregnant after diagnosis, 18 presented with tumor progression during pregnancy or within 8 weeks of delivery [9]. Similarly, Peeters et al. retrospectively studied 52 pregnancies in 50 women diagnosed with glioma and reported an increase in the imaging growth rates during pregnancy in 87% of cases and clinical deterioration in 38% of cases known before pregnancy, while seizures resolved after delivery in 57.2% of these cases [43]. Of note, authors supported that tumors with a higher grade of malignancy, negative expression of alpha-internexin, or positive expression for p53 were associated with tumor progression during pregnancy [43].

The concurrence of pregnancy and glioma is therefore rare, and the management is complicated by the competing priorities of maternal treatment and fetal safety. In the existing literature, mainly single case reports have been published (Table 1), demonstrating primary surgical treatment of the tumors in order to minimize the mass effect and the intracranial pressure, to allow the patient to continue the pregnancy until fetal maturity. A recent Italian survey [44], which included 31 pregnant women diagnosed with a brain tumor during pregnancy, reported that in 18 neurosurgical centers an emergency surgical procedure was required in only 12.9% of the cases. Authors proposed that in patients showing neurological stability, surgical treatment should be delayed, ideally until delivery, while in the case of acute deterioration, pregnancy termination should be considered [44,45]. In our case, the size of the recurrent tumor upon the initial ICU admission was considered small (Figure 2), while the danger for the fetus from a potential surgical or radiological treatment was considered high. Hence, no oncologic treatment was proposed. Fifteen days later, the patient appeared with brain herniation (Figure 4), and craniectomy was considered futile.

### 4.2. Ethics of Pregnancy Support in a Brain-Dead Woman

The brain death definition and the neurological criteria for the diagnosis of BD were first reported by the Ad Hoc Committee of the Harvard Medical School in 1968, after Christiaan Barnard had performed the first human heart transplantation in 1967 [46]. BD was described as the irreversible cessation of all brain functions, defined by the persistence of four signs: complete abolition of consciousness and of any movements, abolition of cranial nerves reactivity, abolition of spontaneous respiration (absence of respiratory movements in the presence of hypercapnia), and flat electroencephalogram (EEG) [46]. Since then, most countries worldwide have adopted these criteria, mainly to permit the withdrawal of unnecessary vital support after a BD diagnosis and organ donation for transplantation [47,48].

In Greece, the definition and criteria for the determination of BD have been legally accepted since 1985 [49]. According to Greek law, after the BD has been determined, the patient is declared dead and is disconnected from the mechanical ventilator, since it is considered unethical and futile to continue supporting vital organ function after the diagnosis of BD [49]. However, the legal framework regarding prolonged maternal somatic support to obtain fetal viability has not been clarified, while there are limited data for prognosis and clinical guidance [50]. Therefore, although our patient had abolished brainstem reflexes upon the second day of ICU admission, we avoided apnea testing until the infant’s delivery day. This way, we overcame possible legal lacunas that could influence healthcare insurance policy.

The progress in intensive care medicine over the last decades has brought a unique challenge in which prolonged corporeal support of BD-pregnant women is possible, until a viable fetus can be delivered [33]. Articles describing maternal somatic support in case of BD during pregnancy have gradually increased over the years. Mostly, these are single-patient case reports or brief literature reviews.

Boran et al. [23] presented a case of a BD pregnant woman and reviewed 24 more cases published between 1982 and 2018 in the English medical literature. The cause of BD was intracerebral hemorrhage in 15 of 24 cases (62.5%), traumatic brain injury in three (12.5%), meningitis in two (8.33%), cerebral venous sinus thrombosis in one (4.17%), and malignant tumor in one case (4.17%) [23]. Later, Dodaro et al. [50] reported the first systematic review of the literature that focused on perinatal outcomes after somatic support in BD pregnant women. The authors included 34 cases of BD mothers. The main cause for BD was intracerebral hemorrhage (68%), while only one of 34 cases (3%) was diagnosed with a cerebral tumor [50]. That was the same case of cerebral tumor that Boran et al. [23] included in their study [50]. That unique case was a 26-year-old woman, at 15 weeks of pregnancy, who developed BD due to an intracerebral hematoma because of a newly diagnosed metastatic melanoma [23]. The basic difference with the case we present is that their patient had a history of a melanoma nine years before the pregnancy; thus, it was considered completely recovered. The brain metastasis was diagnosed upon hospital admission, at 15 weeks of pregnancy [23]. In our case, although the patient and her family were fully informed about the astrocytoma’s prognosis, they refused to undergo all necessary tests for possible fetal karyotypic abnormalities, including a nuchal translucency scan.

The predominant ethical principle for decision-making is autonomy, meaning the capacity for self-determination [51]. After extensive discussions with the couple, after the first ICU admission, and with the husband and other family members, after she deteriorated and was readmitted in ICU, they seemed to realize the severity of the situation and the risks for the pregnancy. However, they insisted on the mother’s corporeal support, hoping for the delivery of a live baby. Nevertheless, patient autonomy is not unlimited. Ethical dilemmas can arise in cases of medical futility. The “benefit” of a medical treatment is often based on a quality-of-life analysis rather than on biological effectiveness [51]. In this case, involving a mother with an end-stage brain carcinoma, in the beginning of the second trimester (16th week of gestational age) and a small-for-gestational-age infant with oligohydramnios, according to the first ultrasonographic biophysical profile upon ICU admission, one might be tempted to decline the requested support as medically futile. However, with respect for the family members’ wishes and with the lack of objective evidence of futility, a decision to proceed with somatic support was made by the ICU team members, since it was believed that the baby could have a chance for survival.

The decision to continue somatic support of a pregnant woman after a diagnosis of BD is both a controversial ethical and legal issue. There are no specific legal regulations associated with somatic support after BD is diagnosed during pregnancy in Greece, and a wide range of options have been reported. The International Federation of Gynecology and Obstetrics has published guidelines for the management of the social and ethical challenges presented by BD during pregnancy [51]. These guidelines recommend that women have the right to die in dignity [51]. Secondly, questions regarding maintaining pregnancy must be answered in consultation with the remaining family members [51]. In the case we present, both the mother and the rest of the family were involved in the decision-making process from the very beginning.

Decisions on delivery in the case of BD should be made according to fetal viability, and surveillance of fetal well-being should be implemented [51]. In our case, the fetus was not viable at the time the mother was neurologically deteriorated, although no signs of fetal damage were found when it was assessed by an obstetrician. Fourthly, there is no low gestational age limit for the onset of fetal rescue after maternal BD [51]. The gestational age of our fetus was estimated to be around 16 weeks upon the second ICU admission, which was low. Both doctors and family members realized the very low probability of a successful delivery of a healthy baby and the risk of potentially fatal complications for the fetus. However, we agreed that this would be the unique chance for the baby to survive.

The interest of the fetus must be the primary consideration, and efforts should be made to promote the birth of a mature, brain-intact infant [51]. This is why, during the whole ICU hospitalization of our patient, every effort was made to secure the stability of the fetus. Finally, the fetus should be allowed to die in utero if maternal or fetal distress calls for immediate delivery with a high probability of an unfavorable outcome [51]. To address this issue, the medical team assessed the level of organ support of the brain-dead mother daily, taking precautions not to provide organ support beyond the level usually provided for BD organ donors; e.g., the use of excessive vasopressors to maintain adequate blood pressure or renal replacement therapy if acute renal failure develops. According to these guidelines, our medical decisions were focused primarily on the safety of the fetus, e.g., the choice of antibiotic treatment.

### 4.3. The Management of the Pregnant Brain Death

Only individual cases of prolonged corporeal support of BD pregnant women have been reported. Thus, there are no guidelines for the medical management of such cases. The physiological support of the pregnant BD/DNC patient could be compared with the management of the BD/DNC organ donor, although it is much more prolonged in the case of pregnancy [52,53,54,55]. In our case, the management was based on the experience we had with BD non-pregnant potential organ donor patients. For specific subjects, such as hypopituitarism, we reviewed the literature. The long-term management of BD/DNC patients may be associated with complications, such as diabetes insipidus, hypopituitarism, hemodynamic instability, cardiac arrhythmias, systolic myocardial dysfunction, disseminated intravascular coagulation, thermoregulatory dysfunction, hypoxemia, and infections [33].

In the case we present, to preserve pulmonary support, the patient was maintained on prophylactic mechanical ventilation, with a tidal volume of 4–6 mL/kg of ideal body weight and a minimum PEEP of 5 cm H_2_O, to prevent ventilator-induced lung injury, barotrauma, and atelecto-trauma [56]. The target oxygen saturation was above 95% and carbon dioxide tension was in the normal pregnancy range of 28 to 32 mm Hg in order to prevent fetal hypercapnia and hypoxemia [57,58]. Despite the necessity for prolonged ventilatory support, tracheostomy was not performed to avoid possible complications due to potential perioperative hemodynamic instability, hypoxemia, bleeding, or infection [59].

Furthermore, the combination of diabetes insipidus and the loss of sympathetic tone contributed to profound hemodynamic instability, which we managed with hemodynamic monitoring-guided crystalloids infusion, hormone replacement, and vasopressors [60]. The primary vasopressor drug we used was vasopressin [61]. Incidentally, vasopressin was compared with norepinephrine during periods of sepsis [62,63]. We tried to minimize the use of norepinephrine in order to prevent its potential negative effects on placental perfusion [64].

The hypothalamic–pituitary axis disruption after brain death determination was treated with hormone replacement therapy, with thyroid hormone for hypothyroidism, corticosteroids for adrenal insufficiency, and vasopressin for diabetes insipidus, while we frequently monitored serum levels of thyroid hormones, serum sodium, and urine output. Of note, data supporting a choice of hormone therapy, dosage, and effect on neonatal outcomes in BD pregnant women are lacking [33].

Moreover, the monitoring of infections and treatment with appropriate antibiotics is imperative to avoid the development of disseminated infections during a long ICU stay. Hyperthermia [65] and hemodynamic instability may delay fetal growth [64], leading to demise if not successfully treated [66,67]. In the case we present, the choice of antimicrobial agents was made considering the cultures’ results and the safety of the individual antibiotics during pregnancy [68].

Finally, special attention was paid to ensure adequate nutrition with mineral elements and vitamins, aiming at the maturation of the fetus. However, the growth of the fetus was gradually delayed. Upon the second ICU admission of the pregnant woman, the fetus was at the 10th percentile of the intrauterine growth curves for the 16th week of gestational age. During the 11-week corporeal support of the mother, the fetus presented intrauterine growth restriction, and, finally, a small-for-gestational-age (only 549 g) neonate was delivered at 27 weeks [69,70].

### 4.4. Obstetric Outcome in Brain Death

Even though the literature in the past has shown reasonable outcomes of infants of BD mothers, there is likely publication bias falsely elevating the survival rates, as cases with poor outcomes are typically less likely to be published. Moreover, delivery of a premature baby that died in neonatal ICU due to multiple complications [50] should not be considered as “successful delivery of a baby”.

We present a case of an unsuccessful delivery of a viable infant of a BD woman at 27 weeks of gestation, since the baby was delivered with a low APGAR score, intubated and mechanically ventilated, and died after 4 weeks in the neonatal ICU due to intracerebral hemorrhage because of prematurity. In a systematic analysis of 30 similar cases published from 1982 to 2010, the mean gestational age at the time of delivery was 29.5 weeks, and only 12/30 infants were viable and survived the neonatal period [71]. Later, in a second brief review of 24 cases published between 1982 and 2018, the mean gestational age at the time of delivery was also 29.05 weeks, and the reported outcome included 7/24 (29.1%) intrauterine deaths and 1/24 (4.16%) death, 1 month after the delivery day [23]. Similarly, Dodaro et al. announced that the mean age of infant delivery was 27.2 weeks, with the infant survival rate ranging from 50% when the gestational age of the delivery was less than 24 weeks to 100% at the age of 28–31 weeks [50].

In a healthy pregnancy, the minimum duration required for the birth of a normal and healthy baby is 32–34 gestational weeks. Before the 24th gestational week, the survival chance of the newborn is 20–30%, with a possibility of a serious neurological disorder of 40%. From 24 to 28 weeks, survival increases to 80%, with a risk of neurological complications at around 10%. From the 32nd week of pregnancy, the survival rate of the fetus rises to 98%, with the risk of neurological complications lower than 2% [72]. Neonatal disability is, among others, proportional to the gestational age of BD diagnosis and delivery. The chance of survival of a baby born in gestational week 22 is 1% and rises to 44% in gestational week 25. The probability of survival without a disability at 30 months shows a similar course and is approximately 0.7% in gestational week 22 and 23% in gestational week 25. From gestational week 32, the survival rate rises to 98%, with a risk of neurological complications lower than 2% [72,73]. With these data in mind, our aim of maternal corporeal support was for the fetus to reach the 32nd week [23].

Moreover, the estimated 180-day survival probability for a neonate with 500–749 gr birth weight was 0.817 (confidence interval 0.799–0.834) [74]. Dodaro et al. also reported that the rate of live birth differed by gestational age at diagnosis of BD [50]. When BD was diagnosed between the 14th and 19th week, the rate of live birth was only 54.5% [50]. In addition, the gestational age is directly proportional to the survival of the neonate, and thus the goal for gestational age at delivery is above 32 weeks [33,50].

It is of note that our patient was at a high risk for chromosomal or structural anomalies of the developing fetus due to her medical history of radiotherapy and chemotherapy. Although there were no relevant ultrasound findings, indicating a possible abnormality, no special prenatal genetic screening was performed due to the family’s wishes. However, a gradual intrauterine growth restriction was observed that could be multifactorial. All complications of BD that have been described could be implicated in fetal restriction, affecting the mother and the placenta [26].

This case highlights multiple medical, legal and ethical issues concerning pregnancy in an end-stage brain cancer patient and also brain death during pregnancy. Taking into account the limited literature so far, it emphasizes aspects related to extended critical care support of a BD patient due to a malignant disease, in order to successfully conclude a pregnancy. As ongoing medical and technical progress will inevitably bring more cases of pregnancy in the context of a BD mother to medical attention, further research is necessary to guide the appropriate and multidisciplinary support of both the mother and the fetus/newborn.

## 5. Conclusions

Pregnancy in end-stage brain cancer patients is an extremely rare but increasingly common condition, and its management is associated with medical, ethical, and legal uncertainties and controversies. When brain death is determined, and a will for maternal corporeal support is expressed, a meticulous multidisciplinary challenge emerges. The extended multiorgan support of the BD mother, in order to prolong the pregnancy, can maximize the chances for the neonate’s survival. However, the possibility that such pregnancies will result in premature deliveries, with multiple immediate or long-lasting complications, is not rare. Hence, both involved parties should be informed with clarity throughout the period of care to ensure support and avoid crises in the interprofessional care team.

## Figures and Tables

**Table 1:** Cases with astrocytoma in pregnancy.

Authors	Country of Origin	Study Population	Brain Lesion	Time of Tumor Diagnosis	Intervention	Complications	Gestational Age of Neonatal Birth	Outcome
Al-Rasheedy IM et al. Neurosciences (Riyadh) 2015;20(4):388–391 [20]	Kingdom of Saudi Arabia	36-year-old woman	GBM	11th gestational week	Chemotherapy and radiation	The mother died 2 weeks post-delivery	28th week with elective cesarean section	Alive infant (890 gr), Apgar scores:8/1, 9/10
Kamata K et al. JA Clin Rep 2017;3(1):18 [11]	Japan	30-year-old woman	Glioma grade III	21st gestational week	Surgical treatment	No complications were mentioned	35th week	Alive infant
Shahraki AD et al. Adv Biomed Res 2019;8:54 [12]	Iran	32-year-old woman, 19 weeks pregnant	Astrocytoma grade I	<1 year before pregnancy	Surgical treatment + radiotherapy	Thrombocytopenia, severe proeclampsia	36th week	Alive infant
Bayley JC et al. World Neurosurg 2019;127:58–62 [21]	Texas, USA	27-year-old woman	Optic pathway gliomas	3rd semester	Surgical treatment post-delivery	No complications were mentioned	31st week	Alive infant
Miyazaki T et al. Case Rep Obstet Gynecol 2019;2019:8340437 [22]	Japan	26-year-old woman	Diffuse midline glioma with H3-K27M mutation	19th week pregnant	Ventriculoperitoneal shunt at 21st gestational week and the symptoms were improved	Intratumoral hemorrhage on postpartum day 2, died 3 weeks later	38th week	Alive infant
Singh P et al. Anticancer Res 2020;40(6):3453–3457 [10]	Dallas, Texas, USA	5 pregnant women with gliomas	1 grade II astrocytoma, 1 grade III anaplastic astrocytoma, 3 grade IV GBM	4/5 during pregnancy (mean 17.25th week), 1/5 prior to pregnancy	5/5 were surgically treated, 1/5 plus radiotherapy	No complications were mentioned	Mean 37th week	5/5 alive infants
Filippi V et al. BMJ Case Rep 2021;14(4):e242135 [13]	Switzerland	34-year-old woman	Diffuse astrocytoma	28th gestational week	Surgical treatment	No complications were mentioned	37th week	Alive infant
Gunasekaran A et al. Clin Neurol Neurosurg 2022;216:107218 [14]	South Carolina, USA	24-year-old woman	GBM grade IV	12th gestational week	Surgical treatment + radiotherapy	No complications were mentioned	34th week	Alive infant
López SO et al. Cureus 2023;15(5):e39016 [15]	Lima, Peru	34-year-old woman	Diffuse astrocytoma	18th gestational week	Surgical treatment	No complications were mentioned	37th week	Alive infant
Venkitasubramony V et al. Front Oncol 2025;15:1700845 [4]	Tampa, Florida, USA	34-year-old woman, 18 weeks pregnant	Anaplastic astrocytoma grade III	10 years earlier	Surgical treatment	No complications were mentioned	26th week	Alive infant
***Pregnancy in brain-dead patients with brain tumor***								
Boran ÖF et al. J Relig Health. 2020;59(6):2935–2950 [23]	Turkey	21-year-old woman	Intraventricular hemorrhage due to mass of unknown origin	19 weeks	6 weeks	VAP	25 weeks	Intrauterine death of the fetus
**Abbreviations:** ARDS: Acute Respiratory Distress Syndrome, GBM: glioblastomas, ICH: IntraCerebral Hematoma, VAP: Ventilator-Associated Pneumonia.								

**Table 2:** Cases of brain death in pregnancy.

Authors	Country of Origin	Study Population	Brain Lesion	Gestational Age at Diagnosis of BD	Duration of Corporeal Support	Complications	Gestational Age of Neonatal Birth	Outcome
Woderska A et al. Ann Transplant 2012;17(1):113–116 [31]	Poland	40-year-old woman, 21 weeks pregnant	SAH	23rd week	1 week	Hemodynamic instability, diabetes insipidus, infections, respiratory failure	24 weeks	Alive infant
Said A et al. Int J Crit Illn Sci 2013;3(3):220–224 [35]	United Arab Emirates	35-year-old woman, 16 weeks pregnant	Intracranial hemorrhage	18th week	110 days	Hemodynamic instability, diabetes insipidus, hypernatremia, pulmonary, CNS and urinary infections, panhypopituitarism	32 weeks	750 gr alive infant Apgar score: 6, 7, 9 at 1, 5 and 10 min
Kinoshita Y et al. Acute Med Surg 2014;2(3):211–213 [27]	Japan	32-year-old woman, 20 weeks pregnant	Cardiac arrest, postanoxic encephalopathy	22nd week	12 weeks	Not mentioned	34 weeks	Alive infant
Wawrzyniak J. Anaesthesiol Intensive Ther 2015;47(1):40–44 [32]	Poland	30-year-old woman, 20 weeks pregnant	SAH due to raptured brain aneurysm	20th week	7 weeks	Infections, massive bleeding from the airways	27 weeks	Alive infant
Holliday S et al. Heart Lung 2017;46(5):397–400 [29]	USA	36-year-old woman, 22 weeks pregnant	Cardiac arrest, postanoxic encephalopathy	23rd week	90 days (started before BD was confirmed)	Hemodynamic instability, diabetes insipidus, infections, respiratory failure	32 weeks	Alive infant
Gopčević A et al. Int J Obstet Anesth 2017;32:82–86 [34]	Croatia	36-year-old woman, 21 weeks pregnant	ICH	21st week	8 weeks	Not mentioned	29 weeks	Alive infant
Pikto-Pietkiewicz I et al. J Crit Care Med (Targu Mures) 2019;5(3):111–114 [36]	Poland	27-year-old woman, 13 weeks pregnant	SAH due to raptured brain aneurysm	Not mentioned with clarity (around 15th week)	100 days	Hemodynamic instability, diabetes insipidus, infections, acute pancreatitis	28 weeks	Alive infant
Reinhold K et al. BML Case Rep 2019;12(9):e231601 [37]	Germany	28-year-old woman, 9 weeks pregnant	trauma	10th week	20 weeks + 4 days	Hypopituitarism, Recurrent bacterial pulmonary and urinary infections, acute pre-eclampsia	30 + 4 weeks	Alive infant
Gal R et al. Am J Case Rep. 2021;22:e930926 [33]	Czeck Republic	27-year-old woman, 16 weeks pregnant	ICH due to rupture of an arteriovenous malformation	16th week	16 weeks	Hemodynamic instability, diabetes insipidus, infections	33 weeks	Alive infant
Warren A et al. J Intensive Care Soc 2021;22(3):214–219 [25]	United Kingdom	26-year-old woman, 19 weeks pregnant	Asthma attack	Not mentioned with clarity	13 weeks	Respiratory failure	32 weeks	Alive infant
Moguillansky N et al. Cureus 2023;15(8):e44172 [24]	Gainesville, USA	31-year-old woman, 22 weeks pregnant	SAH	22nd week	11 weeks	Infections	33 weeks	Alive infant
Cerqueira L et al. Case Rep Obstet Gynecol 2025;2025:2746188 [30]	Portugal	26-year-old woman, 18 weeks pregnant	Asthma attack—cardiac arrest, postanoxic encephalopathy	19th week	100 days	Respiratory failure	32 weeks	Alive infant
Ghio M et al. Transplant Proc 2025;57(1):94–96 [38]	New Orleans, Louisiana, USA	33-year-old woman, 38 weeks pregnant	Anoxic brain injury	38th week	A few days	Fetus death	38 +couple of days	Nonviable fetus
***Pregnancy in brain-dead patients with brain tumor***								
Boran ÖF et al. J Relig Health. 2020;59(6):2935–2950 [23]	Turkey	21-year-old woman, 19 weeks pregnant	Intraventricular hemorrhage due to mass of unknown origin	19th week	6 weeks	VAP	25 weeks	Intrauterine death of the fetus
**Abbreviations:** ARDS: Acute Respiratory Distress Syndrome, BD: Brain Death, CNS: Central Nervous System, Coagulation, GB: Glioblastomas, SAH: SubArachnoid Hemorrhage, VAP: Ventilator-Associated Pneumonia.								

## Data Availability

The data included in this article are not publicly available due to participant confidentiality and privacy agreements. Anonymized data may be available upon reasonable request to the corresponding author.
